# The Co-inhibitor BTLA Is Functional in ANCA-Associated Vasculitis and Suppresses Th17 Cells

**DOI:** 10.3389/fimmu.2019.02843

**Published:** 2019-12-10

**Authors:** Kai Werner, Sebastian Dolff, Yang Dai, Xin Ma, Alexandra Brinkhoff, Johannes Korth, Anja Gäckler, Hana Rohn, Ming Sun, Jan Willem Cohen Tervaert, Pieter van Paassen, Andreas Kribben, Oliver Witzke, Benjamin Wilde

**Affiliations:** ^1^Department of Nephrology, University Hospital Essen, University of Duisburg-Essen, Essen, Germany; ^2^Department of Infectious Diseases, University Hospital Essen, University of Duisburg-Essen, Essen, Germany; ^3^Division of Rheumatology, Department of Medicine, University of Alberta, Edmonton, AB, Canada; ^4^Section of Nephrology and Immunology, Department of Internal Medicine, Maastricht University Medical Center, Maastricht, Netherlands

**Keywords:** ANCA vasculitis, BTLA, co-inhibition, immune checkpoint, Th17 cells

## Abstract

**Objectives:** The activation and inhibition of T-cells has been well-studied under physiological conditions. Co-inhibition is an important mechanism to keep effector T-cells in check. Co-inhibitors mediate peripheral self-tolerance and limit the immune response. Dysfunctional co-inhibition is associated with loss of T-cell regulation and induction of autoimmunity. Therefore, we investigated the co-inhibitor B- and T-Lymphocyte attenuator (BTLA) in ANCA-associated vasculitis (AAV).

**Methods:** Fifty-six AAV patients and 32 healthy controls (HC) were recruited. Flow cytometry was performed to investigate the expression of BTLA on T-cells. Double negative T-cells were defined as CD3^+^CD4^−^CD8^−^. To assess the functionality of BTLA, CFSE-labeled T-cells were stimulated in presence or absence of an agonistic anti-BTLA antibody. In addition, impact of BTLA-mediated co-inhibition on Th17 cells was studied.

**Results:** AAV patients in remission had a decreased expression of BTLA on double negative T-cells (CD3^+^CD4^−^CD8^−^). On all other subtypes of T-cells, expression of BTLA was comparable to healthy controls. TCR-independent stimulation of T-cells resulted in down-regulation of BTLA on Th cells in AAV and HC, being significantly lower in HC. Co-inhibition via BTLA led to suppression of T-cell proliferation in AAV as well as in HC. As a result of BTLA mediated co-inhibition, Th17 cells were suppressed to the same extent in AAV and HC.

**Conclusion:** BTLA expression is altered on double negative T-cells but not on other T-cell subsets in quiescent AAV. BTLA-induced co-inhibition has the capacity to suppress Th17 cells and is functional in AAV. Thus, BTLA-mediated co-inhibition might be exploited for future targeted therapies in AAV.

## Introduction

Anti-neutrophil cytoplasmatic antibody (ANCA)-associated vasculitis (AAV) is an autoimmune disease characterized by the presence of autoantibodies directed against myeloperoxidase (MPO) or Proteinase-3 (PR3) expressed by neutrophils (1). AAV mainly affects small- to medium- sized vessels. T-cells have an important role in the pathogenesis of AAV and persistent T-cell activation is frequently observed (2, 3). Th17 cells and effector memory T-cells are expanded which appears to be independent of disease activity (2, 4, 5). T-cell infiltrates are frequently found in affected organs (6–9). Moreover, defective function of regulatory T-cells has been demonstrated in AAV indicating disturbed immune regulation (10, 11). T-cells are not only restrained by regulatory cell subsets but are also controlled by a system of co-stimulation (12). Positive co-stimulation promotes T-cell activation whereas co-inhibition limits and suppresses activation of T-cells (12). Co-inhibitory signals may induce anergy or cell death of T-cells (12, 13). These mechanisms are important to maintain immune tolerance. Dysfunctional co-stimulation and co-inhibition promote the break of tolerance and are associated with autoimmunity. In human, a defective co-inhibitory PD1/PDL-1 axis is associated with a number of autoimmune diseases such as systemic lupus erythematosus and AAV (14, 15). In addition, targeted blockade of this system promotes the development of AAV (16). The B- and T-Lymphocyte Attenuator (BTLA) is an Ig superfamily member molecule and interacts with the herpes virus entry mediator (HVEM), a member of the TNFR family (13, 17, 18). BTLA is a co-inhibitor which is predominantly expressed on B-cells, T-cells and dendritic cells. BTLA ligation with HVEM results in a reduction of T-cell proliferation (13, 17, 18). Mice with BTLA deficiency show increased T-cell activation and higher levels of circulating TNF-α, IFN-γ, IL-2, and IL-4 (19, 20). However, BTLA as a co-inhibitor is scarcely studied in human autoimmune diseases and its role in disease pathogenesis is unclear. Therefore, it was our goal to examine the expression as well as function of BTLA on T-cells and B-cells in AAV.

## Materials and Methods

### Patients

We enrolled 56 AAV patients who visited the outpatient clinic of the department of Nephrology at the University Hospital Essen. Eleven patients were measured twice. Forty-eight patients were in remission at the time of sampling, eight patients suffered from active vasculitis. The mean Birmingham vasculitis activity score of the active patients was 10 ± 3. None of the active patients were treatment naïve at the time of sampling; all patients had already received low dose steroids. One patient had received one dose of rituximab 2 days before sampling and one treatment cycle with plasma exchange. None of the active patients had received cyclophosphamide recently or before sampling. Two patients suffered from new-onset disease, the remaining six patients had a relapse. The clinical and laboratory characteristics of the quiescent patients at the time of sampling are given in Table 1. As a control cohort, we enrolled 32 persons [18 men and 14 women with median age of 51 (47–54) years] who had no history of immunological, infectious, rheumatic, or malignant disease. The Watts criteria were used to differentiate between GPA, EGPA and MPA (21). EULAR-criteria were used to classify the stage of diseases (22). All patients provided written informed consent. The study was approved by the local institutional review board.

**Table 1:** Clinical characteristics of AAV patients in remission.

**Total, *n***	**48**
Age, median (IQR), years	55 (19–84)
Gender, female/male, *n*	24/24
PR3/MPO/neg, *n*	25/22/1
Disease duration, median (IQR), months	36 (2–236)
Localized/systemic disease, *n*	12/36
CMV anti-IgG, +/−/na, *n*	20/15/13
Prednisone +/−, *n*	42/6
Azathioprine +/−, *n*	19/29
Mycophenolate Mofetil +/−, *n*	19/29
Methotrexate +/−, *n*	1/47
Rituximab +/−, *n*	2/46
Leflunomide +/−, *n*	1/47

*na, not available; neg, negative*.

### Flow Cytometric Analysis of T-Cells and B-Cells

Separation of peripheral blood mononuclear cells (PBMC) out of whole blood was performed by Ficoll-density gradient centrifugation. PBMC were counted by BLAUBRAND® counting chamber. For surface staining, the following monoclonal antibodies were used: anti-human CD19 (PB, Beckman Coulter, Krefeld, Germany), anti-human CD3 (HORV450, BD Biosciences, Heidelberg, Germany), anti-human CD4 (PerCP, Biolegend, Koblenz, Germany), anti-human CD8 (APC-H7, BD Biosciences), anti-human CD45RA (APC, Beckman Coulter), CD272 (BTLA, PE, Biolegend). Appropriate isotype-controls and fluorescence-minus-one controls were used. After isolation, PBMC were incubated with monoclonal antibodies for 30 min at room temperature in the dark followed by washing steps with PBS. Double negative T-cells were defined as CD3^+^CD4^−^CD8^−^ (DN), naïve double negative T-cells were defined as CD3^+^CD4^−^CD8^−^CD45RA^+^ and memory double negative T-cells were defined as CD3^+^CD4^−^CD8^−^CD45RA^−^. Memory T-helper-cells were defined as CD3^+^CD4^+^CD45RA^−^. The analysis of the samples was carried out on a FACS Navios flow cytometer from Beckman Coulter. Where indicated, PBMC were stimulated by phorbol 12-myristat 13-acetat (PMA) (50 ng/ml, Sigma Aldrich, Taufkirchen, Germany), and Ionomycin (1 μg/ml) (Sigma Aldrich) in RPMI 1640 medium (Gibco Invitrogen, Darmstadt, Germany) supplemented with 10% heat-inactivated fetal calf serum (Greiner Bio-One, Frickenhausen, Germany), 100 U/mL penicillin and 100 μg/mL Streptomycin (both Gibco Invitrogen). PBMC were incubated for 4 h in a 5% CO_2_ atmosphere at 37°C in culture medium. Unstimulated samples served as controls and were incubated without stimulus. After incubation, surface staining was performed and cells were analyzed on a flow cytometer (Navios, Beckman Coulter). Kaluza Analysis Software (Version 1.5, Beckman Coulter) was used for analysis of flow cytometric data.

### Functional Studies of BTLA

B-cells were isolated by using a negative selection method based on a magnetic bead technology (B cell isolation kit II, Miltenyi Biotec, Bergisch Gladbach, Germany), typical purity of isolated B-cells was above 95%. In order to quantify the proliferation of lymphocytes, isolated PBMCs or isolated B-cells were labeled with CFSE. CFSE was used at a concentration of 2 μM (Thermo Fisher Scientific, Dreieich, Germany). PBMC labeled with CFSE were then stimulated with anti-CD3 (0.5 ng/ml, clone HIT3a, Biolegend) and anti-CD28 (0.5 ng/ml, clone 28.2, Biolegend) in the presence or absence of an agonistic anti-BTLA antibody (50 ug/ml, clone MIH26, BioLegend) for 72 h. Isolated B-cells were stimulated with the TLR-agonist CpG ODN 2006 (Invivogen Toulouse, France) in the presence or absence of an agonistic anti-BTLA antibody (50 ug/ml, clone MIH26, BioLegend) for 72 h. In conditions without anti-BTLA antibody, an isotype control was used instead at the same concentration (50 ug/ml, mouse IgG2a, Invitrogen). Incubation was carried out at 37°C in 5% CO_2_ atmosphere. After 72 h, PBMC were stained with anti-CD3 (Pacific Blue, Beckman Coulter), anti-CD4 (APC, Beckman Coulter), anti-CD8 (APC-H7, Becton Dickinson), and 7AAD (Biolegend). B-cells were restimulated with PMA (10 ng/ml, Sigma–Aldrich, Taufkirchen, Germany), Ionomycin (1 μg/ml, Sigma–Aldrich) in the presence of Brefeldin A (5 μg/ml, BD Biosciences) for 6 h followed by surface staining, fixation and permeabilization (CytoFix/CytoPerm kit, BD Biosciences, Erembodegen, Belgium). For intracellular flow cytometric analysis the Breg staining was performed with: anti-CD3(HorV450), anti-CD19(PB), anti-7AAD and IL-10 (APC). After fixation and permeabilization, PBMCs were stained intracellularly for IL-10 (APC) and CD69 (PE-CY7). Appropriate isotype controls were used to confirm specificity of staining and to discriminate background staining. The suppressive capacity of BTLA was determined as the relative inhibition of cell proliferation and was calculated as follows: (proliferated fraction of cells without anti-BTLA [isotype] MINUS proliferated fraction of cells with anti-BTLA) DIVIDED by proliferation of PBMCs without anti-BTLA [isotype] MULTPLIED by 100. In addition, cell culture supernatants were collected. All samples were stored at −20°C until bulk analysis. Human IL-17A and IFN-γ ELISA immunoassays were purchased by R&D systems Europe, Ltd. (Quantikine ELISA). The test was performed according to the manufacturer's instructions. IL-17A and IFN-γ levels are expressed as pg/mL.

### Statistical Analysis

All values are given as mean ± standard error of the mean. The Mann-Whitney *U* test was used to detect statistically significant differences between two unpaired groups. The Wilcoxon test was performed to assess paired groups. *P* < 0.05 were considered as significant. GraphPad Prism 6.0c (GraphPad Software, Inc., California) was used for statistical analysis.

## Results

### Reduced Expression of BTLA on Double Negative T-Cells in AAV

**Figure 1:**
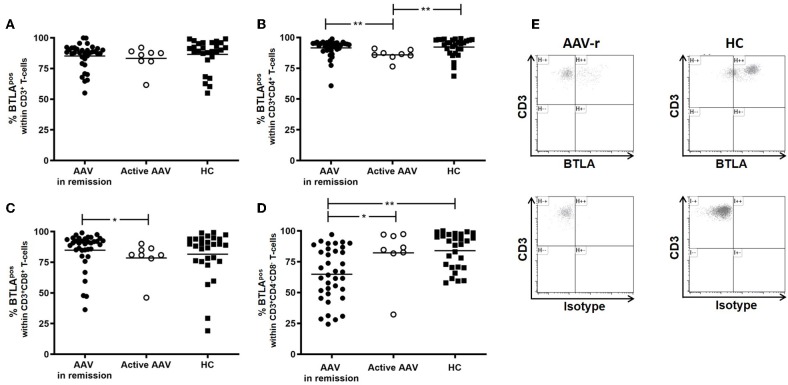
BTLA expression on circulating T-cells in AAV and HC. **(A)** Expression of BTLA was comparable between AAV und HC on CD3^+^ T-cells. **(B)** BTLA expression did not differ on Th cells and on **(C)** cytotoxic T-cells in quiescent AAV vs. HC. Patients with active disease showed diminished BTLA expression on Th cells and cytotoxic T-cells. **(D)** On CD3^+^CD4^−^CD8^−^ T-cells, BTLA was diminished in quiescent AAV as compared to HC. In active patients, BTLA expression was enhanced as compared to patients in remission. **(E)** Representative flow cytometric data is depicted. The plots are gated on CD3^+^CD4^−^CD8^−^ T-cells. Significant differences as calculated by the Mann-Whitney *U*-test are indicated: *p < 0.05, **p < 0.01.

In quiescent AAV patients (AAV-r), the BTLA expression did not differ from HC on peripheral T-cells (AAV-r vs. HC, CD3^+^ T-cells: %BTLA^pos^, 85.2 ± 1.7% vs. 86.6 ± 2.4%, *p* = 0.19, Figure 1). the same was found for T-helper cells (Th cells, AAV-r vs. HC, %BTLA^pos^ within CD3^+^CD4^+^ T-cells: 91.5 ± 1.2% vs. 92.2 ± 1.4%, *p* = 0.21), memory Th cells (AAV-r vs. HC, %BTLA^pos^ within CD3^+^CD4^+^CD45RA^−^ T-cells: 90.1 ± 1.1 vs. 92.3 ± 1.6%, *p* = 0.2), and cytotoxic T-cells (AAV-r vs. HC, %BTLA^pos^ within CD3^+^CD8^+^ T-cells: 84.9 ± 2.5% vs. 81.6 ± 3.7%, *p* = 0.54). On double negative T-cells (DN, CD3^+^CD4^−^CD8^−^) the expression of BTLA was significantly decreased in AAV (AAV-r vs. HC, %BTLA^pos^ within CD3^+^CD4^−^CD8^−^ T-cells: 64.9 ± 3.6% vs. 84.0 ± 2.7%, *p* < 0.001, Figure 1). The lower BTLA expression in AAV-r could also be found on naïve DN T-cells (AAV-r vs. HC, %BTLA^pos^ within CD3^+^CD4^−^CD8^−^CD45RA^+^, *n* = 34/27; 91 ± 1.8% vs. 94 ± 2.1%, *p* < 0.05), and memory DN T-cells (AAV-r vs. HC, %BTLA^pos^ within CD3^+^CD4^−^CD8^−^CD45RA^−^, *n* = 34/27; 67.1 ± 3.4% vs. 85.5 ± 2.9%, *p* < 0.05). The frequency of DN T-cells was comparable between AAV und HC (AAV-r vs. HC, %CD4^−^CD8^−^ within CD3^+^ T-cells: 4.2 ± 0.4 vs. 5.1 ± 0.5%, *p* > 0.05). It was further studied whether the BTLA expression pattern was dependent on disease activity. For this purpose, patients with active ANCA-vasculitis (AAV-a) were recruited. Interestingly, BTLA was downregulated on T-helper-cells in patients with active disease as compared to HC and patients in remission (%BTLA^pos^ within CD4^+^ T-helper-cells, AAV-a vs. HC: 85.9 ± 1.6% vs. 92.2 ± 1.4%, *p* = 0.006; AAV-a vs. AAV-r: 85.9 ± 1.6% vs. 91.5 ± 1.2%, *p* = 0.001). Cytotoxic T-cells showed reduced BTLA expression in active patients when compared to patients in remission (%BTLA^pos^ within CD8^+^ T-cells: 78.6 ± 4.8% vs. 84.9 ± 2.5%, *p* = 0.02). In contrast, BTLA was upregulated on DN T-cells in active disease as compared to quiescent disease (%BTLA^pos^ within DN T-cells, 82.2 ± 7.5% vs. 64.9 ± 3.6%, *p* = 0.03). BTLA expression seemed to be dependent on disease activity and was differentially expressed on the specific T-cell subsets.

### Longitudinal Assessment of BTLA Expression on T-Cells in AAV

**Figure 2:**
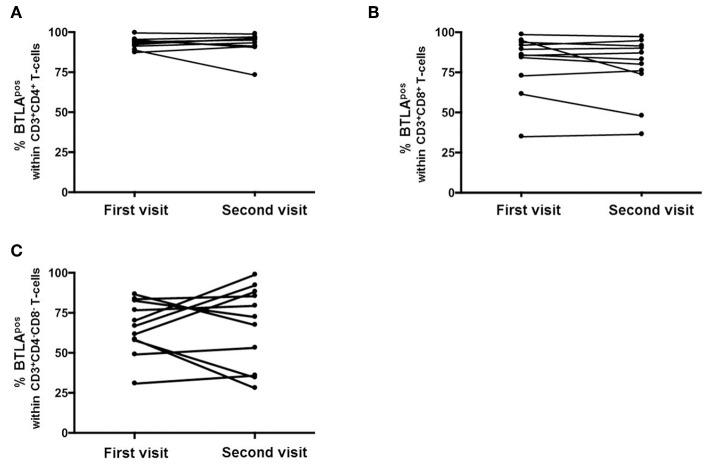
Longitudinal assessment of BTLA expression on T-cells. The expression of BTLA on Th cells **(A)**, cytotoxic T-cells **(B)**, and DN T-cells **(C)** was comparable at the first vs. the second visit.

To detect variability of BTLA expression, eleven AAV-r patients were measured twice over a period of 1 year (Figure 2). In AAV patients, the expression of BTLA did not change significantly between the first and the second visit on Th cells (AAV-r patients at the first visit vs. second visit, 93.1 ± 3.3% vs. 95.1 ± 6.9%, *p* = 0.7) and on cytotoxic T-cells (AAV-r patients at the first visit vs. second visit, CD3^+^CD8^+^ T-cells: %BTLA^pos^, 85.1 ± 18.6% vs. 83 ± 19.4%, *p* = 0.41). On double negative T-cells, the variability of BTLA expression was not significantly altered (AAV-r patients at the first visit vs. second visit, CD3^+^CD4^−^CD8^−^: %BTLA^pos^, 66.7 ± 16.7% vs. 72.4 ± 25.2%, *p* = 0.76).

### BTLA Expression Is Elevated on Stimulated T-Cells in AAV

**Figure 3:**
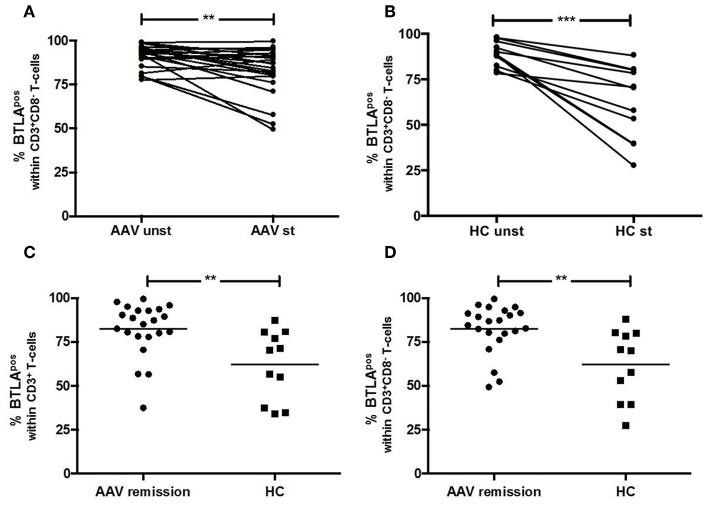
BTLA expression on T-cells after TCR-independent stimulation. **(A,B)** BTLA was decreased on CD3^+^CD8^−^ T-cells after stimulation with PMA/Ionomycin. **(C,D)** After stimulation, the expression of BTLA was significantly more reduced on T-cells derived from HC as compared to AAV patients. Significant differences as calculated by the Wilcoxon-test for paired samples are indicated as ***p < 0.001. Significant differences as calculated by the Mann-Whitney *U* test for unpaired samples are indicated: **p < 0.0024.

BTLA expression decreased after stimulation on CD3^+^ T-cells in AAV-r (AAV-r before stimulation vs. after stimulation, CD3^+^ T-cells: %BTLA^pos^, 91.3 ± 1.3% vs. 82.3 ± 2.9%, *p* < 0.05, Figure 3) and in HC (HC before stimulation vs. after stimulation, CD3^+^ T-cells: %BTLA^pos^, 88.9 ± 2.1% vs. 62.2 ± 6.1%, *p* < 0.05). CD3^+^CD8^−^ Th cells also showed decreased expression of BTLA after stimulation in AAV-r (AAV-r before stimulation vs. after stimulation, CD3^+^CD8^−^ T-cells: %BTLA^pos^, 91.3 ± 1.4% vs. 82.3 ± 3.1%, *p* < 0.05, Figure 3) and in HC (HC before stimulation vs. after stimulation, CD3^+^CD8^−^ T-cells: %BTLA^pos^, 88.9 ± 2.1% vs. 62.2 ± 6.1%, *p* < 0.05). In direct comparison, BTLA expression was more reduced on T-cells in HC after stimulation. This applied to the CD3^+^ (AAV-r vs. HC, CD3^+^ T-cells: %BTLA^pos^, 82.3 ± 3.3% vs. 62.3 ± 6.0%, *p* < 0.05) and the CD3^+^CD8^−^ (AAV-r vs. HC, CD3^+^CD8^−^ T-cells: %BTLA^pos^ 82.3 ± 2.9% vs. 62.2 ± 6.1%, *p* < 0.05) T-cell population. Likewise, the ratio of BTLA expression (BTLA expression on stimulated CD3^+^ T-cells divided by BTLA expression on unstimulated CD3^+^ T-cells) was increased in AAV compared to HC (AAV-r vs. HC, BTLA ratio on CD3^+^ T-cells: 0.89 ± 0.003 vs. 0.71 ± 0.06, *p* < 0.05; AAV-r vs. HC, BTLA ratio on CD3^+^CD8^−^ T-cells: 0.9 ± 0.03 vs. 0.69 ± 0.06, *p* < 0.05), indicating a less pronounced downregulation of BTLA in AAV patients.

### BTLA Suppresses T-Cell Proliferation and Th17 Cells

**Figure 4:**
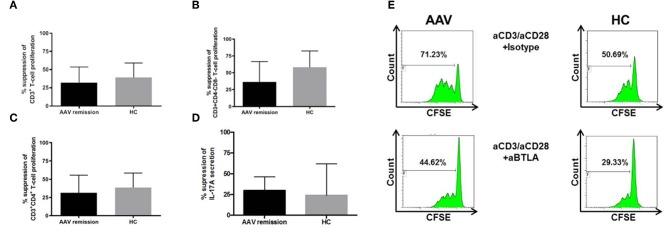
Functional assessment of BTLA. CFSE-labeled PBMC were stimulated by anti-CD3/anti-CD28 (each 0.5 ng/ml) in presence of agonistic anti-BTLA (50 ug/ml) or isotype (mouse IgG2a, 50 ug/ml). Proliferated fraction was determined by CFSE dilution. IL-17A levels were determined from cell culture supernatants. **(A)** T-cell proliferation was inhibited by agonistic anti-BTLA treatment in AAV und HC. **(B)** Likewise, Th cell and **(C)** DN T-cell proliferation was suppressed. **(D)** Th17 cells were inhibited by agonistic anti-BTLA treatment as indicated by reduced IL-17A secretion. Suppression was similar between AAV patients and HC. IL-17A levels were determined from cell culture supernatants. **(E)** Representative flow cytometric data is shown. Plots are gated on viable CD3^**+**^ T-cells. Suppression was calculated as follows: [proliferated fraction of T-cells/cytokine levels without anti-BTLA [isotype] MINUS proliferated fraction of T-cells/cytokine levels with anti-BTLA] DIVIDED by proliferation of PBMC without anti-BTLA [isotype] MULTIPLIED by 100. Data is depicted as mean ± standard deviation.

The function of BTLA was examined in 17 AAV-r patients and 10 HC. CFSE labeled PBMC were stimulated with anti-CD3/CD28 in the presence and absence of an agonistic anti-BTLA antibody. Stimulation of T-cells in presence of an agonistic anti-BTLA antibody resulted in suppression of T-cell proliferation in AAV and HC (relative inhibition of T-cell proliferation in %, AAV vs. HC, 32.1 ± 5.2% vs. 39.0 ± 6.3%, *p* = 0.33, Figure 4). Th cells (relative inhibition of Th cell proliferation in %, AAV vs. HC, 31.4 ± 5.9% vs. 38.63 ± 6.3%, *p* = 0.36, Figure 4) and cytotoxic T-cells (relative inhibition of the cytotoxic T-cell proliferation in %, AAV vs. HC, 32.1 ± 5.1% vs. 28.5 ± 7.9%, *p* = 0.81) were suppressed to the same extent in AAV and HC. Interestingly, the proliferation of DN T-cell was also suppressed by BTLA-induced co-inhibition (relative inhibition of the T-cell proliferation in %, AAV-r vs. HC, 36.4 ± 7.4% vs. 58.2 ± 9.1%, *p* = 0.11). In both groups, BTLA-mediated suppression inhibited IL-17A secretion (AAV-r vs. HC, suppression of IL-17A secretion, 29.9 ± 5.0% vs. 24.1 ± 14.3%, *p* = 0.9, Figure 4). The levels of INF-γ secretion were reduced by BTLA-mediated suppression in AAV and HC (AAV-r vs. HC, suppression of INF-γ secretion, 63.2 ± 3.4% vs. 74.2 ± 8.1%, *p* = 0.17).

### Role of BTLA Expression on B-Cells in HC and Patients

**Figure 5:**
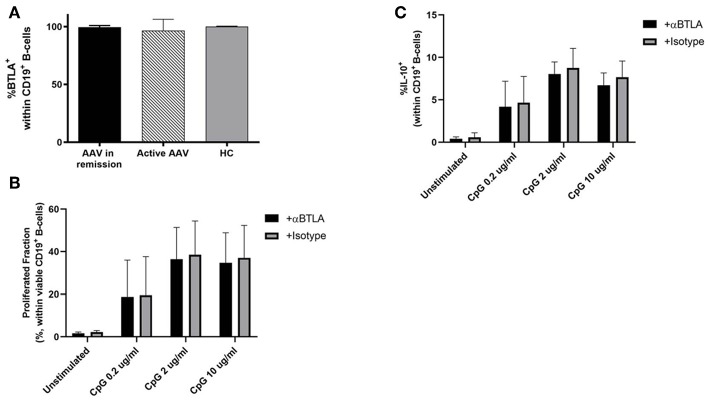
BTLA expression and function on B-cells. **(A)** Expression of BTLA was comparable between AAV und HC on CD19^+^ B-cells. **(B,C)** CFSE-labeled isolated CD19^**+**^ B-cell from HC were stimulated by CpG ODN 2006 for 72 h in presence of agonistic anti-BTLA (50 ug/ml) or isotype (mouse IgG2a, 50 ug/ml). Proliferated fraction was determined by CFSE dilution, IL-10 production was determined by intracellular flow cytometry after restimulation. Agonistic anti-BTLA treatment did not suppress B-cell proliferation. IL-10 production by regulatory B-cells was not affected by agonistic anti-BTLA treatment. Data is depicted as mean ± standard deviation.

The BTLA expression pattern was analyzed on B-cells in HC (*n* = 16), patients in remission (AAV-r, *n* = 27) and patients with active disease (AAV-a, *n* = 8, Figure 5). The fraction of BTLA expressing B-cells was comparable between patients in remission, active patients, and HC (%BTLA^pos^ within CD19^+^ B-cells, AAV-r vs. AAV-a: 99.53 ± 0.3% vs. 96.45 ± 3.5%, *p* = 0.63; HC vs. AAV-r: 99.95 ± 0.04% vs. 99.53 ± 0.3%, *p* = 0.2; HC vs. AAV-a: 99.95 ± 0.04% vs. 96.45 ± 3.5%, *p* = 0.13, Figure 5). Furthermore, the functional role of BTLA on B-cells was assessed (Figure 5). For this purpose, isolated B-cells from HC were stimulated *ex vivo* in presence or absence of agonistic anti-BTLA. B-cell proliferation was assessed by CFSE dilution. Moreover, IL-10 production by B-cells was determined to assess whether agonistic anti-BTLA treatment hampers the function of anti-inflammatory regulatory B-cells. Agonistic anti-BTLA treatment did not suppress B-cell proliferation. Even sub-optimal stimulation in presence of agonistic anti-BTLA did not result in a significant inhibition of CpG-induced B-cell proliferation. Moreover, differentiation of regulatory B-cells and IL-10 production were not suppressed by activation of BTLA (Figure 5). Thus, in contrast with the findings on T-cells, B-cell activation and regulatory B-cell differentiation were not susceptible to agonistic anti-BTLA treatment suggesting control by other co-inhibitory pathways.

### Association of BTLA and Clinical Parameters: BTLA Expression on DN T-Cells Correlates With Relapse Rate

Further analyses were performed to unravel associations of BTLA expression with clinical parameters. Interestingly, relapse rate was associated negatively with BTLA expression on DN T-cells (*r* = −0.3, *p* = 0.04). There was no association of relapse rate with BTLA expression on CD4^+^ T-helper-cells or CD8^+^ cytotoxic T-helper-cells. Thus, increased BTLA expression on DN T-cells correlated with better disease control and outcome. In addition, patients were stratified by history of biopsy proven renal involvement (RI) to study whether BTLA expression differs between these two groups. There was no difference comparing BTLA expression between both patient groups (with RI vs. without RI, %BTLA^pos^ within CD4^+^ T-cells: 91.86 ± 1.4% vs. 90.59 ± 1.9%, *p* = 0.2; %BTLA^pos^ within CD8^+^ T-cells: 86.53 ± 2.3% vs. 79.91 ± 7.3%, *p* = 0.5; %BTLA^pos^ within DN T-cells: 67.89 ± 3.9% vs. 55.86 ± 7.9%, *p* = 0.2). Likewise, renal function at the time of measurement -determined as glomerular filtration rate (GFR) estimated by CKD-EPI formula- did not correlate with BTLA expression on T-cell subsets (CD4^+^ T-cells: *r* = 0–16, *p* = 0.34; CD8^+^ T-cells: *r* = −0.15, *p* = 0.38; DN T-cells: *r* = 0.14, *p* = 0.4). Furthermore, it was assessed if differences can be detected between patients with MPO-ANCA as compared to patients with PR3-ANCA. Patients were stratified by antibody-status at time of diagnosis. There was no difference with regard to BTLA expression on T-cell subsets (PR3 vs. MPO patients, %BTLA^pos^ within CD4^+^ T-cells: 90.53 ± 1.7% vs. 93.13 ± 1.2%, *p* = 0.3; %BTLA^pos^ within CD8^+^ T-cells: 86.53 ± 2.4% vs. 83.11 ± 5.3%, *p* = 0.6; %BTLA^pos^ within DN T-cells: 68.25 ± 3.8% vs. 59.59 ± 7.0%, *p* = 0.33). It was further studied whether treatment impacted BTLA expression. Thus, cumulative cyclophosphamide (CYC) dosage and steroid dosage at time of sampling were correlated with BTLA expression on T-cell subsets. A significant positive correlation was found regarding BTLA expression on DN T-cells and cumulative CYC dosage (*r* = 0.36, *p* = 0.04); there was no association between cumulative CYC dosage and BTLA expression on T-helper-cells or cytotoxic T-cells (CYC/%BTLA on CD4^+^ T-cells: *r* = 0.09, *p* = 0.6; CYC/%BTLA on CD8^+^ T-cells: *r* = −0.14, *p* = 0.42). Steroid dosage at time of sampling was not significantly associated with BTLA expression on CD4^+^, CD8^+^, or DN T-cells (CD4^+^ T-cells: *r* = −0.12, *p* = 0.5; CD8^+^ T-cells: *r* = 0.32, *p* = 0.06; DN T-cells: *r* = 0.09, *p* = 0.6).

## Discussion

The expression of the negative co-stimulator BTLA was diminished on double negative T-cells in AAV-r and correlated with disease activity as well as relapse rate. BTLA expression was unaltered on Th-, cytotoxic -T-cells, and B-cells in quiescent AAV. After stimulation with PMA and Ionomycin, BTLA expression persisted in AAV and was downregulated in HC. The co-inhibition of T-cells via BTLA during TCR-mediated stimulation led to suppression of T-cell proliferation and inhibited secretion of IL-17 as well as INFγ. Thus, the BTLA axis seems intact and functional in AAV.

T-cell regulation is an essential feature of a healthy immune system (23). T-cell regulation is driven by negative co-stimulation mediated via a number of different systems such as the CTLA4-axis, BTLA-axis and PD1-axis (24). The CTLA4- and the PD1-axis have both been well-studied and the importance for maintaining immune tolerance has been shown (12, 13, 15, 25). In addition, both co-stimulatory systems are blocked for therapeutic purposes in malignant diseases to boost immune responses. BTLA has been investigated in experimental models. Otsuki et al. showed that BTLA ligation transmits an inhibitory signal to T-cells and thus might play an important role in T-cell tolerance (26). Krieg et al. noticed that the stimulation of murine T-cells in presence of an agonistic BTLA antibody results in decreased IL-2 production and diminished occurrence of CD25^+^ T-cells (27). There is evidence from animal studies that BTLA knockout leads to autoimmune diseases (20, 28, 29).

In our study, we found that BTLA expression on Th cells, cytotoxic T-cells and B-cells was comparable between quiescent patients and HC when assessed under basal conditions. Interestingly, the expression of BTLA was significantly decreased on double negative T-cells in AAV-r. This was found on naïve DN- and on memory DN T-cells. BTLA expression on DN T-cells correlated with disease activity as well as relapse rate indicating a probable role in disease pathogenesis. Altered expression of other co-inhibitory molecules such as CTLA4 and PD-1 on T-cells has been reported in AAV. Wilde et al. found that the expression of PD-1 on T-helper was increased on Th cells from AAV patients as compared to HCs (15). Steiner et al. could show that expression levels of CTLA-4 were significantly increased on CD4^+^ and on DN T-cells in AAV (25). After stimulation by PMA and Ionomycin, the CTLA-4 levels were increased on T-cells derived from HC but T-cells from AAV patients had an impaired response (25). Ye et al. also found a decreased expression of BTLA in Behcet's disease on Th cells and this was associated with an abnormal Th17 and Th1 immune response (30). In a recent publication by Sawaf et al., expression of BTLA on T-cell subsets was comparable in patients with SLE and HC (31). However, despite this finding, the authors showed that BTLA functionality was significantly impaired in SLE patients.

DN T-cells are poorly studied yet, but it is known that these cells are expanded in systemic lupus erythematosus and that their relative proportion correlates with disease activity (32). In patients with lupus nephritis, DN T-cell showed pro-inflammatory features producing IL-17A and were found in renal lesions (33). In Sjogren's disease, DN-T-cells have been identified as the cell population that is primarily involved in the production of IL-17A and plays an important role in the maintenance of inflammatory processes (34).

In contrast, in murine models of acute kidney injury, DN were found as tissue-resident anti-inflammatory T-cell population in acute kidney injury (35). In our study, the relative proportion of circulating DN T-cells was comparable between AAV and HC. The aberrant expression of BTLA on DN T-cells in AAV could nevertheless disrupt the co-inhibitory function and thereby contribute to systemic- and renal inflammation. Furthermore, we found that after stimulation of PBMC with PMA and Ionomycin, BTLA was downregulated in HC. Downregulation was less pronounced in AAV. Han et al. showed that BTLA was upregulated in mice after TCR-mediated stimulation (36). Sedy et al. have also shown in a mouse model that the expression of BTLA was variable after stimulation via TCR (37).In contrast to these studies, we stimulated T-cells in a TCR-independent manner, possibly explaining the different expression pattern. Data on human T-cells being stimulated short-term with PMA/Iono is lacking. Less downregulation of BTLA on T-cells in AAV may cause an increased susceptibility to BTLA-mediated suppression which my counterbalance persistent T-cell activation.

Next, we further tested the function of the BTLA-axis. Suprisingly, B-cell proliferation and differentiation of regulatory B-cells were not susceptible to treatment with agonistic anti-BTLA. There is conflicting data on the functional meaning of BTLA expression on B-cells. HVEM, a cognate ligand of BTLA, has been demonstrated to suppress B-cell proliferation (38). In support of our own data, another study failed to show a suppressive effect on B-cell proliferation when using agonistic anti-BTLA treatment (39). In contrast, Co-inhibition of T-cells with an agonistic anti-BTLA antibody suppressed anti-CD3/-CD28 induced proliferation of HC and AAV patients to the same extent. Thus, the BTLA pathway appears to be intact in AAV. As mentioned above, Sawaf et al. found defective functionality of BTLA in SLE patients (31). T-cell stimulation via TCR in presence of agonistic anti-BTLA was less efficient suppressing proliferation and CD25 upregulation of T-cells in SLE patients as compared to HC (31). It is conceivable that other autoimmune diseases harbor a different pattern of functional co-inhibitors as multiple, different and redundant co-inhibitory systems exist to control T-cell immunity. Therefore, it is not surprising that another co-inhibitor, the PD1/PDL-1 pathway, is reported to be dysfunctional in AAV (15).

From our data, we also gain important novel information on the physiological role of BTLA. Agonistic treatment of TCR stimulated T-cells reduced not only proliferation –as has been reported previously- but also suppressed IL-17A and INFγ secretion in HC as well as in patients. Thus, BTLA-induced suppression seems to impact effector T-cells such as Th1 and Th17 cells efficiently. IL-17A is a key factor in pathogenesis of AAV and IL-17A knock out protects from disease in murine models (3, 40). As BTLA ligation led to suppression of *ex vivo* stimulated Th17 cells in HC and AAV, this co-inhibitor might be exploited in future for therapeutic purposes. Similar approaches have already been tested in experimental animal transplant models and agonistic anti-BTLA treatment conferred protection from allograft rejection (41, 42). However, it has not been unraveled whether agonistic BTLA treatment also counteracts established tissue inflammation by regulating lesional T-cells. Regulating circulating vs. lesional T-cells has important implications. If lesional T-cells are not functionally regulated by BTLA, the therapeutic potency of targeting BTLA might be limited to prophylactic purposes such as remission maintenance. It is a limitation of our study that we did not investigate lesional T-cells. However, we were focused on the role of BTLA in circulating T-cells to unravel whether this co-inhibitory pathway is in principle functional in ANCA-vasculitis. As the access to lesional T-cells is very limited in human disease and may not allow functional studies, the role of BTLA in regulating lesional T-cells could be addressed in future by employing one of the animal models available for ANCA-vasculitis.

In summary, the BTLA axis seems functional and intact in AAV. As BTLA ligation suppresses Th17 cells efficiently, this pathway should be investigated further as potential therapeutic target in AAV.

## Data Availability Statement

The datasets generated for this study are available on request to the corresponding author.

## Ethics Statement

The studies involving human participants were reviewed and approved by the local ethics committee of the University Hospital Essen. The patients/participants provided their written informed consent to participate in this study.

## Author Contributions

KW designed the study, performed the experiments, performed data analysis, and wrote the manuscript. SD, YD, AB, MS, and XM performed the experiments, performed data analysis, and wrote the manuscript. JK, AG, and HR performed data analysis and wrote the manuscript. JC, PP, and AK designed the study and wrote the manuscript. OW and BW designed the study, performed data analysis, and wrote the manuscript.

### Conflict of Interest

The authors declare that the research was conducted in the absence of any commercial or financial relationships that could be construed as a potential conflict of interest.
